# Sparse Zero-Sum Games as Stable Functional Feature Selection

**DOI:** 10.1371/journal.pone.0134683

**Published:** 2015-09-01

**Authors:** Nataliya Sokolovska, Olivier Teytaud, Salwa Rizkalla, MicroObese consortium, Karine Clément, Jean-Daniel Zucker

**Affiliations:** 1 Institute of Cardiometabolism and Nutrition, ICAN, Assistance Publique Hôpitaux de Paris, Pitié-Salpêtrière Hospital, Paris, France; 2 Sorbonne Universités, UPMC University Paris 6, UMR_S 1166, ICAN, NutriOmics Team, Paris, France; 3 INSERM, UMR S U1166, NutriOmics Team, Paris, France; 4 LRI, University Paris 11, INRIA Saclay, Orsay, France; 5 Research Institute for Development, UMI UMMISCO, Bondy, France; Kyushu University, JAPAN

## Abstract

In large-scale systems biology applications, features are structured in hidden functional categories whose predictive power is identical. Feature selection, therefore, can lead not only to a problem with a reduced dimensionality, but also reveal some knowledge on functional classes of variables. In this contribution, we propose a framework based on a sparse zero-sum game which performs a stable functional feature selection. In particular, the approach is based on feature subsets ranking by a thresholding stochastic bandit. We provide a theoretical analysis of the introduced algorithm. We illustrate by experiments on both synthetic and real complex data that the proposed method is competitive from the predictive and stability viewpoints.

## 1 Introduction

Feature selection is a problem which arises naturally in a number of applications, and, in particular, in biomedical tasks, where the number of parameters is potentially very high but just a small subset of them is informative. In the past decades a number of model selection methods have been proposed, including methods for group and hierarchical feature selection, which are supposed to reveal some structure of underlying data. An important issue is the stability of feature selection methods. A result of model selection is very sensitive to the samples used during feature extraction and to a learning method. Moreover, the power of statistical feature selection methods was hardly studied. Recently, [1] considered 32 state-of-the-art feature selection approaches, however, a simple univariate *t*-test appeared to reach the highest stability and a very reasonable performance.

It has been observed that functions captured by different feature sets can be very similar, despite a very low degree of overlapping between these feature sets. In our contribution, we tackle this complex problem of stable feature selection on the functional level. We hope that our results will provide biologists with some intuition on functional classes of features, i.e., on functional classes of biological parameters.

Our research is motivated by real rich high-dimensional biomedical applications, in particular, by challenges of quantitative metagenomics and transcriptomics. In quantitative metagenomics we study the collective genome of the micro-organisms inhabiting human body, and, since recently, it has become feasible to measure the abundance of bacterial species. Scientists doing pre-clinical research are often interested to find groups of bacterial species associated with a particular phenomenon. So, e.g., in [2] the challenge is to associate groups of bacterial species with the development of obesity. Another source of data, transcriptomics considers the complete set of RNA transcripts produced by the genome at different time points, and allows scientists to analyze various phenomena in various tissues. The metagenomics and transciptomics applications are extremely high-dimensional, and to extract the most significant biomarkers, some efficient feature selection is needed. The number of features can be very high but at the same time the features are structured in unobserved functional categories of comparable predictive performance, i.e., there are subsets of features that encode the same biological function and therefore perform equally.

It is known [3, 4] that there exist strong correlations or similarities between genes which can be examined, for instance, with an automated annotation tool. In our work, we consider both stability on the level of features and on the level of their functions.

To select the most pertinent subsets of parameters and to reach the stability on the functional level, we propose to apply sparse zero-sum games. We introduce a thresholding stochastic bandit which efficiently ranks sets of features. The idea to use multi-armed bandits to carry out ranking, is not new. To our knowledge, the first attempt to rank with a bandit algorithm was done by [5], and it was applied to Web documents ranking; [6] applied the ranking to digital libraries. An approach which is in some sense similar to ours was proposed by [7], where the problem of feature selection is formalized as a reinforcement learning problem (a one-player game), and the learning procedure relies on the Monte-Carlo tree search.

One of the most classical games considered by the game theory, is the prisoner’s dilemma which allows to investigate pairwise cooperations. In its original form, there are two players in the game, and the players can either cooperate or defect. Both players get the reward *R* if they cooperate and they get the punishment *P* if both defect. If one player defects and another cooperates, the first one obtains the temptation *T*, and the second the payoff *S*, where *T* > *R* > *P* > *S*. The game’s strong Nash equilibrium is the situation where both players defect, i.e. the situation from which each player could only do worth by changing its move.

Various extensions of the prisoner’s dilemma have been proposed. In particular, the problem how to promote the evolution of cooperation has been studied. For instance, it has been shown that in a spatial social dilemma game, where players can be represented as nodes on a grid, cooperators can profit through forming clusters of cooperators which are protected from exploitation of defectors [10]. In [11] it has been shown that it is important to define the highest reputation neighbors as strategy donors to promote cooperation. Such clusters of collaboration promoters are robust, and protect efficiently against defectors.

Another important factor which influences cooperative behavior is inertia. [12] demonstrated that cooperation with small inertia will be easily invaded by defectors, and with large inertia the cooperation level will hardly change since the players are not likely to change their strategies. The study [12] discusses an intermediate inertia level which leads to an optimal cooperation.

Public good games come from experimental economics. Players decide how many of their private tokens should be put into a public pool. Then the “public good” is multiplied by a factor and is evenly divided among all players. The group’s reward is maximized when all players contribute all their tokens. However, the Nash equilibrium is zero contributions by players.

A spatial version of the game is defined on a grid where each agent is a node, has several neighbors, and plays simultaneously with all the neighbors. As a result, one observes spatial clusters of cooperation and defection. It was noticed that changing existing links between neighbors can be beneficial for the cooperation, as well as growth of the network has been observed [13]. An aspiration-induced version of the game was recently proposed [14] where a player would cut the link with the neighbor if his payoff from the group centered on this neighbor does not exceed an aspiration level. In this case, the player will connect to another randomly selected player. It has been shown that there exists an aspiration level which induces an optimal cooperation.

In spatial public good games it is usually assumed that the investment of all players is identical. [15] considers the case where players are heterogeneous, what is much more realistic. The study concludes that heterogeneous investments can promote cooperation.

To our knowledge, our contribution is the first attempt to see the problem of group feature selection as a sparse matrix game. Without loss of generality, a stochastic bandit used in our experiments, is the EXP3 algorithm [8, 9]. We illustrate by our experiments that the proposed framework achieves both a stability and performance which are not worse, and in some cases are better, than ones of the state-of-the-art methods.

The novel thresholding bandit learns a sparse probability distribution on sets of features. This is identical to estimating sparse Nash equilibria. Finding Nash equilibria of games in strategic form, is a challenging task, especially if games are large-scale. In our contribution we consider bimatrix (two-player) zero-sum games, and refer to them, for short, as “matrix games”. The proposed approach combines both advantages: compact mixed equilibria and fast computation. We illustrate both by artificial and real biomedial applications that the introduced framework can be applied for the biomarker discovery from high-dimensional data.

This paper is organized as follows. Section 2 discusses the sparsity issues in large games, and how to induce sparsity on mixed Nash equilibria. In Section 3 we present the thresholding stochastic bandit for model selection. We provide some theoretical analysis of the introduced method in Section 3.1. In Section 4 we discuss some similarity and stability measures which we use to evaluate the feature selection methods. In Section 5 we show the results of our experiments on simulated and real data sets. Concluding remarks and perspectives close the paper.

## 2 Sparsity in Zero-Sum Matrix Games

In this section, we consider matrix games and the state-of-the-art methods to find Nash and *ϵ*−Nash equilibria of these games.

We are interested in particular in the situation where the mixed strategies played by agents are sparse. The intuition behind such a sparse mixed strategy is as follows. Although the number of all possible pure strategies can be very big or even infinite, the number of reasonable moves can be quite small. Speaking more formally, sparse mixed strategies are related to games with limited randomness: “to play a game” means that a player samples a pure strategy what entails randomness. In real life, due to computational issues, the randomness is limited or not available. [16].

[17] proposed an algorithm to find sparse Nash equilibria which was a building block for Monte-Carlo Tree Search, and the main goal was to accelerate bandits. The very recent work of [18] introduces an algorithm which is similar to [17] and is based on the rounding solution of the EXP3 what leads to better theoretical bounds.

A zero-sum matrix game is usually defined by a payoff matrix *M*. Let *k*_1_ be a number of pure strategies of player 1, and *k*_2_ be a number of pure strategies of player 2. Without loss of generality, let the values of the payoff matrix be in the interval [0, 1]. When player 1 chooses action *i* ∈ ⟦1, *k*_1_⟧ and player 2 chooses action *j* ∈ ⟦1, *k*_2_⟧, then player 1 gets reward *M*_*i*, *j*_ and player 2 gets reward −*M*_*i*, *j*_.

Mixed strategies *x* and *y* of player 1 and player 2 are probability distributions on their pure strategies. A best response *x* of player 1 to the mixed strategy *y* of player 2 is a strategy which maximizes the expected payoff *xMy* of player 1. Similarly, a best response of player 2 maximizes his/her expected gain.

A Nash equilibrium [19] is a pair of mixed strategies that are best responses to each other. More formally, a Nash equilibrium is a pair (x*, y*) with $x^{*}\in {\left[0,1\right]}^{k_{1}}$ and $y^{*}\in {\left[0,1\right]}^{k_{2}}$ such that

$$\begin{array}{c} \forall x^{\prime },y^{\prime },\;x^{\prime }My^{*}\leq x^{*}My^{*}\leq x^{*}My^{\prime }, \end{array}$$ (1)

where the sum of the elements of x* and of y* equals one; this will be implicitly assumed in this paper for maxima or minima over mixed strategies.

An *ϵ*-Nash equilibrium [20] is an approximation (*x*, *y*) of a Nash equilibrium such that

$$\begin{array}{c} \forall x^{\prime },y^{\prime },\;x^{\prime }My-\epsilon \leq xMy\leq xMy^{\prime }+\epsilon . \end{array}$$ (2)

This reflects the idea that players do not intend to change their strategies, since the amount of gain they could get by choosing other strategies is very small. Every Nash equilibrium is surrounded by a region of *ϵ*-Nash, and that is why *ϵ*-Nash equilibria always exist [20] for any *ϵ* > 0.

Matrix games can be solved (in the sense that an exact Nash equilibrium can be found) by linear programming (LP) [21]. Linear programming can itself be solved polynomially, with exponent 3.5. Therefore, finding the Nash equilibrium of a matrix game is polynomial. However, this result has two weaknesses:

- the exponent 3.5 is not negligible;
- this result holds for deterministic matrix games only, in which playing a given pair (*i*, *j*) of strategies always leads to the same result; the case of a random payoff (independently and identically drawn from a payoff distribution ${\tilde{M}}_{i,j}$) is not covered.

One of the earliest approaches to find Nash equilibria is an algorithm called fictitious play [22]. It is an iterative method which is simple to implement, however, it is assumed that the players know their payoff matrices. If a game is large and it is not feasible to enumerate all possible strategies, and therefore, store the payoff matrices, the application of the fictitious play in its original form is not possible.

This is why an extensive literature, including bandits algorithms, is devoted to how to find *ϵ*-Nash equilibria, with exponent 1 (as a function of *K* = max(*k*_1_, *k*_2_), within logarithmic factor), with a limited dependency in *ϵ* (exponent 2).

It has been shown in [23] that one can find an *ϵ*-Nash equilibrium of a matrix game in time *O*(*K* log(*K*)/*ϵ* ^2^) with probability at least $\frac{1}{2}$. In particular, [23] has shown that sublinear complexity makes sense for the problem under consideration: we do not have to read the matrix entirely to solve the game approximately. This implies that we measure the complexity on a machine with two crucial components:

- random-access memory, so that we can sample any coefficient of the matrix without reading all elements of the matrix;
- and equipped with a random generator, so that we can randomly shuffle the indices of the strategies (otherwise, there are counter-examples to the complexities, and we will see that randomization is a necessary tool for sublinear complexities in matrix games).

This idea was extended in [24], showing that the same can be done with stochastic problems, i.e., cases where the result of a game is stochastic as a function of the actions: for a pair (*i*, *j*) of actions (action *i* for the first player, action *j* for the second player), the reward is drawn in [0, 1] according to some probability distribution ${\tilde{M}}_{i,j}$ (independently at each trial), with expectation *M*_*i*, *j*_.

In a number of games, there are many unreasonable strategies, which humans would not even consider. Under an unreasonable strategy we mean a strategy which contain suboptimal actions. The number of reasonable strategies is much smaller than the complete set of pure strategies. There are two main reasons for using sparsity in matrix games:

1. find a sparse mixed strategy, so that we can manipulate pure strategies in a computationally efficient manner;
2. find a computationally efficient approach to estimate a Nash equilibrium.

It has been shown by [25] and [26] that an impressive sparsity can be expected from matrix games without any assumption on the matrix. It is possible to find a subset of ⌈log(*K*)/*ϵ* ^2^⌉ strategies (for each player) which is an *ϵ*-approximate Nash equilibrium. The existence of a deterministic algorithm with running polynomial time to find this subset has been proved by [27].

Therefore, we can answer the first question above concerning sparse pure strategies, but not the second one. If we test all subsets of this size to find the solution, the cost is *O*(*K* ^*O*(log(*K*))^) [25]. This cost is higher than the cost of LP for exact solution.

Recently [17, 28], and [29] proposed purification and thresholding techniques to cope with large games. These methods belong to post-processing approaches. The idea of the purification is to choose a pure strategy with highest probability and to play it with probability 1. If there are several pure strategies with the same high probability value, they are played with equal probability. The thresholding of [28] and [29] is less extreme than the purification. Only probabilities of pure strategies which are less than some threshold are put to zero. Our thresholding algorithm is similar to this idea.

Another avenue to increase the computational speed is to use the tree structure. The idea that sequences of choices instead of pure strategies can be used, has been introduced by [30]. The sequences of choices are efficiently represented as a tree. The number of these sequences is linear and not exponential in the size of the game tree. The same idea has been exploited in [31] and [32]. [32] has proposed to find Nash equilibria by a variation of the Lemke-Howson algorithm [33].

However, removing non-dominant strategies can have some unpredictable effects. It has been illustrated on the example of Poker game [34] that space reduction in large games is not obvious, and that a solution for a small game cannot be always easily extrapolated to the initial large game. That is why it is important to control how suboptimal the approximate solutions are.

## 3 Large-Scale Problems: Sparsity via Thresholding Bandits

A Nash equilibrium defined above as Eq (1) is a pair of mixed strategies where non of the players expects a better reward if he changes unilaterally his strategy. Note that it is possible to have several Nash equilibria in a game, however, the value of the game (x*)^*T*^ My* does not change.

Our goal is to find sparse mixed strategies for two players, and to do it efficiently. In this section, we propose an approach which finds compact Nash equilibria. In the following, we consider an *ϵ*−Nash equilibrium which is defined as a pair (*x*, *y*)

$$\begin{array}{c} \underset{y^{\prime }}{\inf}x^{T}My^{\prime }>{\left(x^{*}\right)}^{T}My^{*}-\epsilon , \end{array}$$ (3)

$$\begin{array}{c} \underset{x^{\prime }}{\sup}x^{\prime T}My<{\left(x^{*}\right)}^{T}My^{*}+\epsilon . \end{array}$$ (4)

In our contribution, we do not assume that the matrix *M* is sparse. On the contrary, we suppose that the Nash equilibria and the *ϵ*-Nash equilibria have a very compact support size.

The intuition behind the novel approach is based on that sparsity can be induced via elimination of those strategies, whose estimated probability values are not significant after the number of rounds *t*:

$$\begin{array}{c} x_{i}^{\prime }=\{\begin{array}{c} x_{i}\;\text{if}\;x_{i}>t^{\alpha }/t,\\ 0\;\text{if}\;x_{i}\leq t^{\alpha }/t, \end{array} \end{array}$$ (5)

where *x*_*i*_ is a *i*-coordinate of the probability distribution, $x_{i}^{\prime }$ is a corresponding (sparse) approximation, and parameter *α* ∈ (0, 1). The thresholding algorithm is summarized in Algorithm 1. Note that *x*_*i*_ is associated with a subset of features, and not with one feature.

The probability distribution *x* can be estimated by a bandit algorithm such as INF (implicitly normalized forecasters) or EXP3 (exponential exploration-exploitation) algorithm (see [8] and [24]). A stochastic bandit algorithm, for example, the EXP3 algorithm, drafted as Algorithm 2, can be used to rank the sets of features. In our experiments in Section 5 we will use the EXP3 algorithm.

**Algorithm 1** THRESHOLDING-BANDIT: remove strategies with low weight

Find an approximate solution (*x*, *y*) of the zero-sum matrix game (after *t* iterations)

**for** all pure strategies *x*_*i*_, *y*_*j*_ **do**

**if** *x*_*i*_ < *t* ^*α*^/*t* **then**

$x_{i}^{\prime }=0$

**end if**

**if** *y*_*j*_ < *t* ^*α*^/*t* **then**

$y_{j}^{\prime }=0$

**end if**

**end for**

$x^{\prime }\leftarrow \frac{1}{\sum_{i}x_{i}^{\prime }}x^{\prime }$

$y^{\prime }\leftarrow \frac{1}{\sum_{i}y_{i}^{\prime }}y^{\prime }$

Consider (*x*′, *y*′) to be the approximate Nash equilibrium

Optionally: use the bandit algorithm again, on strategies with non-zero coefficients in *x*′, *y*′ only

Below, we consider the theoretical analysis of the proposed thresholding bandit.

### 3.1 Analysis of the Thresholding Bandit

Here we consider the thresholding procedure, presented as Eq (5) and Algorithm 1, which gives empirically good results but is proved only in the asymptotic case [17]. We have no rate for the thresholding algorithm, but conjecture that it is very strong when there is strong sparsity, possibly with a posteriori re-optimization of the probabilities by a second pass of EXP3 or INF.

**Proposition**. *Consider a game with unique Nash equilibrium with support of size k*′ and $\alpha =\frac{3}{4}$. *Consider Algorithm 1 for choosing x*′, *an approximate Nash equilibrium obtained by truncation of the result of t iterations of INF. Then, for t sufficiently large, x*′ *contains all the K*′ *pure strategies involved in the Nash equilibrium and only them*.

**Algorithm 2** EXP3

Initialize *w*_*i*_(1) = 1, for *i* ∈ {1,…, *n*}

Set *γ* ∈ (0, 1)

**for** *t* = 1,…*T* **do**

$x_{i}(t)=(1-\gamma )\frac{w_{i}}{\sum_{j=1}^{K}w_{j}}+\frac{\gamma }{K}$ for all *i*

 Draw *i*_*t*_ randomly according to *x*_1_(*t*),…, *x*_*K*_(*t*)

**for** *j* = 1,…, *K* **do**

${\hat{r}}_{j}(t)=\left\{ \begin{array}{c} r_{j}(t)/x_{j}(t)\text{, if}\;j=i_{t}\\ 0\text{, otherwise}\; \end{array}\right.$

$w_{j}(t+1)=w_{j}(t)\text{exp}(\gamma {\hat{r}}_{j}(t)/K)$

**end for**

**end for**

**Proof**. Let x* be the distribution on pure strategies for player 1, the Nash equilibrium.

The INF algorithm approximates the Nash equilibrium in the sense that after *t* iterations and with probability at least $\frac{1}{2}$, the output is a *ϵ*-Nash equilibrium with $\epsilon =O(\sqrt{K/t})$.

As the Nash is supposed to be unique and the value *V*(*x*) = inf_*y*_ *x* ^*t*^ *My* of a mixed strategy is piecewise linear and convex, we get ${||x_{t}-x^{*}||}_{\infty }=O(\sqrt{K\;\text{log}(K)/t})$. Therefore, for *t* sufficiently large, one of the two cases occurs for each *i* ∈ [[1, *K*]]:

- (x*)_*i*_ > 0, and then $x_{i}={\left(x^{*}\right)}_{i}+O(\sqrt{K\;\text{log}(K)/t})$;
- or (x*)_*i*_ = 0, and then $x_{i}=O(\sqrt{K\;\text{log}(K)/t})$.

In both cases, for *t* sufficiently large, (*x*_*t*_)_*i*_ < *t* ^*α*^/*t* is equivalent with (x*)_*i*_ = 0. This is the expected result.

## 4 Similarity and Stability Measures

To compare the outcomes of different feature selection methods, we adopt two measures. We also estimate the stability on the functional level. The first measure we apply is a similarity measure [3, 4]. The I-overlap similarity measure [4] between two sets of features 𝓕_1_ and 𝓕_2_ is computed as the number of variables that are present in both sets, ∣𝓕_1_ ∩ 𝓕_2_∣. The value is normalized in such a way that the final result is in the interval [0;1], where the normalization factor is ∣𝓕_1_ ∪ 𝓕_2_∣. The I-overlap is computed by averaging over all pairwise comparisons.

The second measure we test, is a stability measure [35, 36] called relative weighted consistency CW_rel_. This measure is subset-size-unbiased. Let us carry out feature selection *k* times and obtain 𝓢 = {𝓢_1_,…,𝓢_*ω*_} subsets of selected features from *ω* experiments. $\Omega =\sum_{i=1}^{\omega }|{𝓢}_{i}|$ is the sum of cardinalities of sub-sets 𝓢_*i*_ and *F*_*f*_ is the number of times the variable *f* ∈ 𝓕 was observed in 𝓢. The CW measure is defined as follows

$$\begin{array}{c} CW\left(S\right)=\sum_{f\in F}\frac{F_{f}}{\Omega }\frac{F_{f}-1}{\omega -1}. \end{array}$$ (6)

The CW_rel_ measure is obtained by adjusting the CW value by its minimal and maximal values:

$$\begin{array}{c} CW_{\text{rel}}\left(S,F\right)=\frac{CW\left(S\right)-CW_{\min}\left(\Omega ,\omega ,F\right)}{CW_{\max}\left(\Omega ,\omega ,F\right)-CW_{\min}\left(\Omega ,\omega ,F\right)}. \end{array}$$ (7)

As we have already mentioned, biological functions expressed by different gene sets can be similar although the overlap between these sets is very low. To compare the functional overlap of the genes sets, we refer to the Gene Ontology (GO), and we apply the CW_rel_ measure on the functional level.

## 5 Experiments

In this section, we demonstrate the results of our experiments on two artificial data sets, and on a real complex original biomedical application.

A “small *n*, large *p*” scenario is simulated in Section 5.1, where we demonstrate the accuracy and stability of the thresholding bandits in the case where the number of observations is very small compared to the number of parameters. In Section 5.2 we consider the performance of the proposed method on an artificial data where features belong to functional categories. Section 5.3 illustrates our results on a real biomedical challenge which includes several heterogeneous data types.

[1] has recently performed an extensive comparison of stability of more than thirty feature selection methods. The conclusion of the study was that the univariate *t*-test is the most stable method. Therefore, we compare our results to the univariate *t*-test.

On the artificial data, we show that the accuracy and stability of the proposed thresholding bandit is higher than one of the t-test for a case where the number of observations is much less than the number of parameters (Section 5.1). In Section 5.2 we show on a simulated data set, that the thresholding bandit is efficient for subsets feature ranking where features belong to functional categories. Finally, in Section 5.3 we consider a real high-dimensional biomedical application, and illustrate a high accuracy and stability of the introduced method compared to such approaches as t-test, SVM, and a stochastic bandit without thresholding.

### 5.1 A “Small *n*, Large *p*” Scenario

We simulate a “small number of observations *n*, large number of parameters *p*” scenario similar to [36]. We compare the accuracy of the thresholding bandit with the *t*-test feature selection procedure, [36] make also use of the *t*-test to rank features in their experiments. We choose the *n*/*p* ratio to be 0.1, what means that in our data set, the number of parameters is 10 times bigger than the number of observations. The problem is a binary classification, and the observations from two classes are drawn from normal distributions 𝓝(*μ*, *σ* ^2^) and 𝓝(−*μ*, *σ* ^2^), where *μ* is a mean vector, and its cardinality ∣*μ*∣ = *p*; *σ* ^2^ = 1 in our experiments. To simulate not identical forms of the probability density, we apply a degree *γ* to the vector *μ*, and *μ* = *μ* ^*γ*^, where *γ* is fixed to 0.9.

The number of observations in the experiment is fixed to 10, and the number of parameters is fixed to 100 (*n*/*p* = 0.1). We run 200 Monte-Carlo simulations for experiments with different number of selected features; in this experiment we consider cases with 5, 7, 10, and 50 selected features. The number of subsets of features to be explored by the thresholding bandit is 50, and the number of iterations is 2000 (the number of iterations in the EXP3 algorithm).

**Fig 1:**
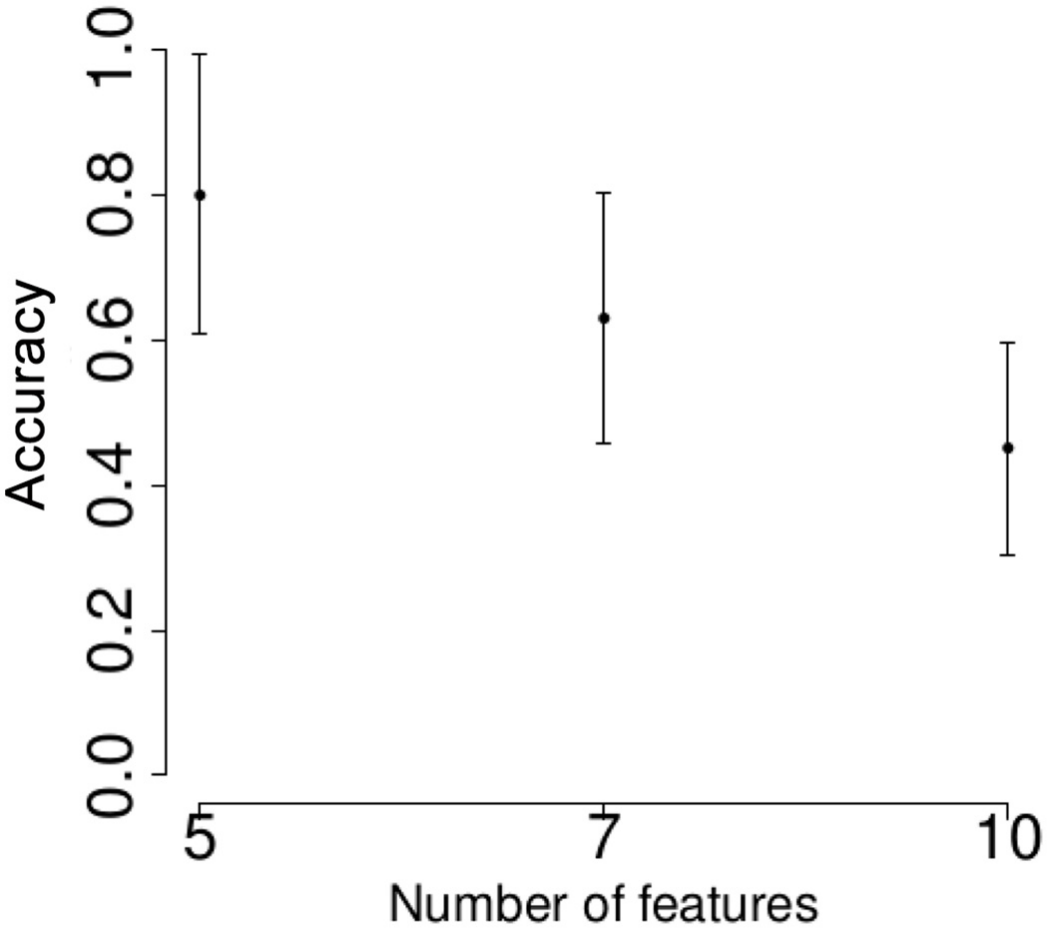
Cross validation accuracy rate for the *t*-test feature selection.

**Fig 2:**
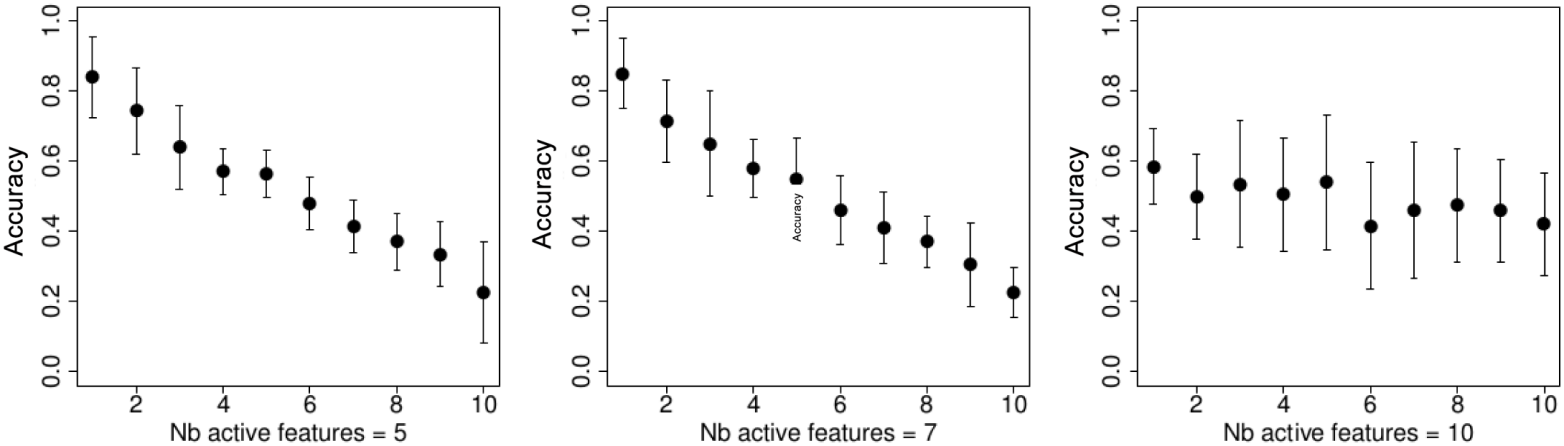
Accuracy of the stochastic bandit (EXP3) ranking as a function of ranked feature sets, the number of feature sets is 10. On the left: the number of active features equals 5; in the center: the number of kept parameters is 7, on the right: the number of selected features is 10.

**Fig 3:**
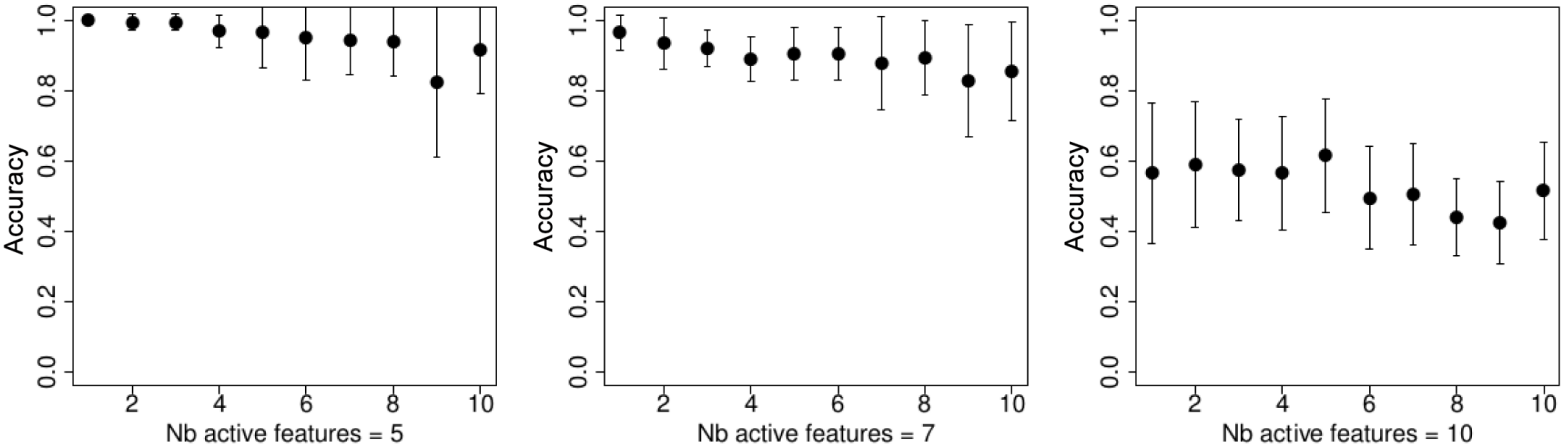
Accuracy of the thresholding bandit as a function of ranked feature sets, the number of feature sets is 10. On the left: the number of active features equals 5; in the center: the number of kept parameters is 7, and on the right: the number of selected features is 10.

**Fig 4:**
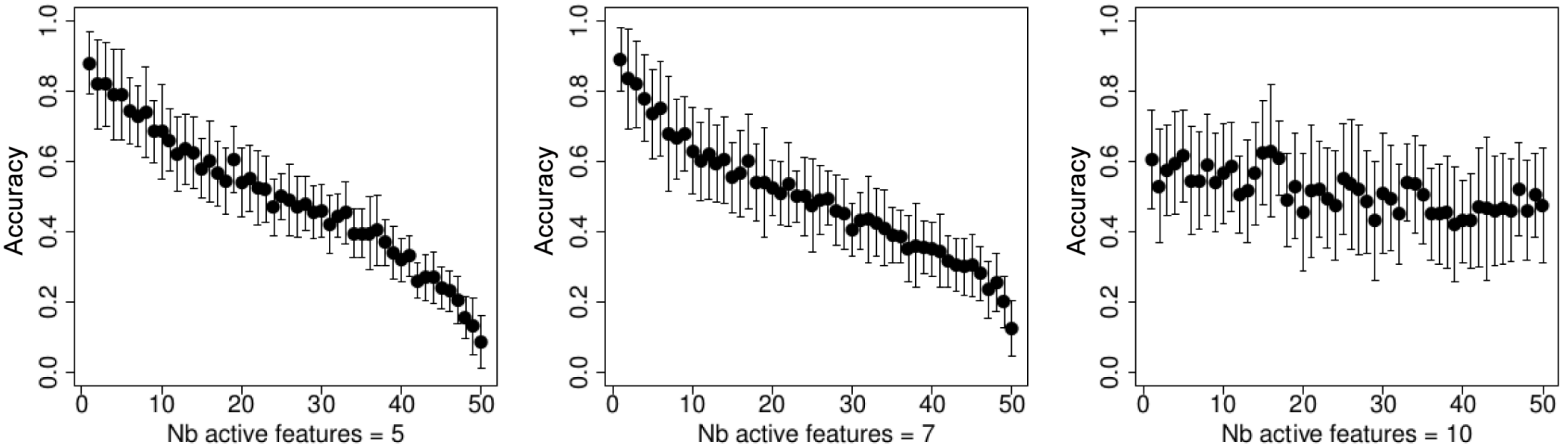
Accuracy of the stochastic bandit (EXP3) ranking as a function of ranked feature sets, the number of feature sets is 50. On the left: the number of active features equals 5; in the center: the number of kept parameters is 7, and on the right: the number of selected features is 10.

**Fig 5:**
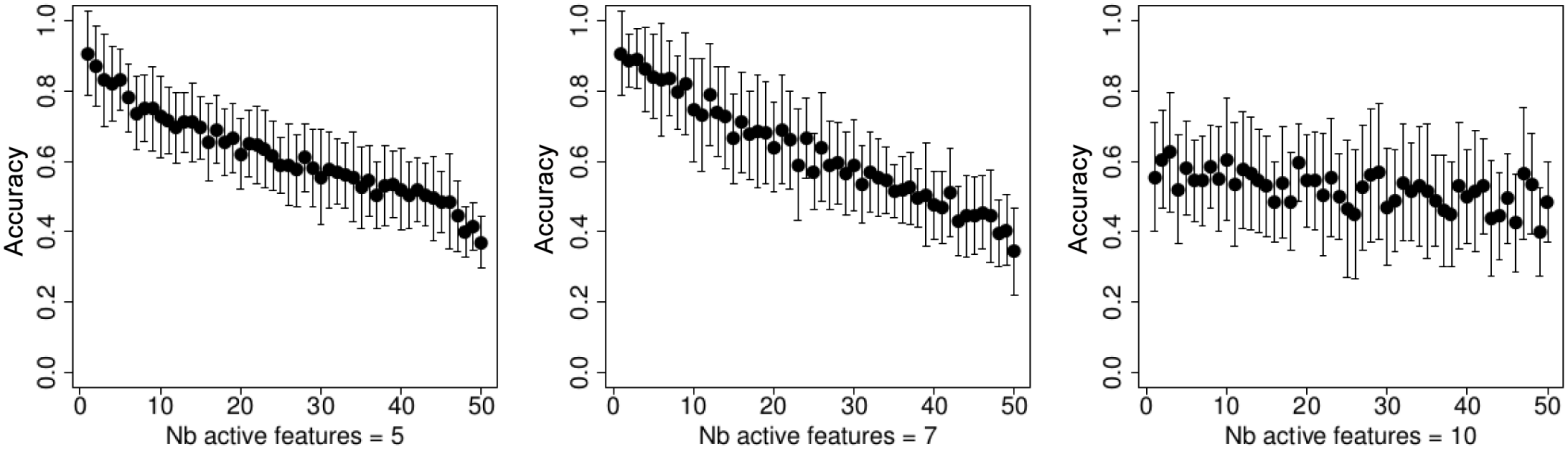
Accuracy of the thresholding bandit ranking as a function of ranked feature sets, the number of feature sets is 50. On the left: the number of active features equals 5; in the center: the number of kept parameters is 7, and on the right: the number of selected features is 10.

On Fig 1 we show the 10-folds cross validation accuracy rate for the *t*-test feature selection. It is easy to see that the best performance is achieved when the number of kept parameters is the smallest, and the accuracy is around 0.8. Figs 2–5 demonstrate the performance for the stochastic bandits methods. Figs 2 and 4 show the accuracy for the ranking by the stochastic bandit without thresholding (EXP3); Figs 3 and 5 show the performance for the thresholding bandit. We plot the accuracy as a function of ranked feature sets. On each plot above on the left: the number of active features equals 5; above on the right: the number of kept parameters is 7, and below the number of selected features is 10. On each plot, we show the accuracies for the strategies of the obtained ranking, so that the first strategy has got the highest probability, and the last one has the lowest probability to be played. Figs 2 and 3 show accuracies for the case where the number of feature sets is 10; Figs 4 and 5 illustrate the performance for 50 feature sets. We observe that the stochastic bandits feature selection method ranks the features set quite well. We also see that the thresholding stochastic bandit outperforms the stochastic bandit, and finds more relevant sets of features.

We examined how stable the feature selection methods are. Table 1 provides some stability values. As a stability measure, we use the relative weighted consistency [35, 36], where the result is close to 0, if the stability is very poor, and close to 1, if the stability is very high. Our results show that, in terms of the stability, the proposed framework is not worse than the method that was reported to be one of the most stable.

**Table 1:** Stability for different number of active features and approaches.

Method	n = 5	n = 7	n = 10
*t*-test	0.05	0.14	0.2
Ranking by EXP3	0.09	0.14	0.3
Ranking by EXP3 with thresholding	0.04	0.14	0.1

### 5.2 Small Scaled Experiment with Functional Categories of Features

In this section, we show our results on a simulated problem, where features belong to functional categories, and where features from the same class or category are functionally redundant. We design a problem, where we have three functional categories or three classes of features. Each class includes three parameters that are considered to be functionally identical, i.e., the total number of parameters is 9. For the optimal prediction, an algorithm should select at least one feature from each class, i.e., three parameters. We draw 15 feature sets (each contains 3 features; the same feature can occur more than once in a feature set). The first three sets are optimal, in other words, they contain parameters from all the functional classes. The feature sets 4–15 are not optimal: the parameters either from one or two functional classes are missing.

The sets of features are ranked as follows. At each round *t* of Algorithm 2 a set of features to be tested is drawn according to its probability. The sets of training and of testing data are also drawn. The accuracy value on the test data is used as the reward in the EXP3, and *γ* = 1/(log(*t* + 1)). The vector of probabilities associated with the feature sets, is updated at each iteration.

**Fig 6:**
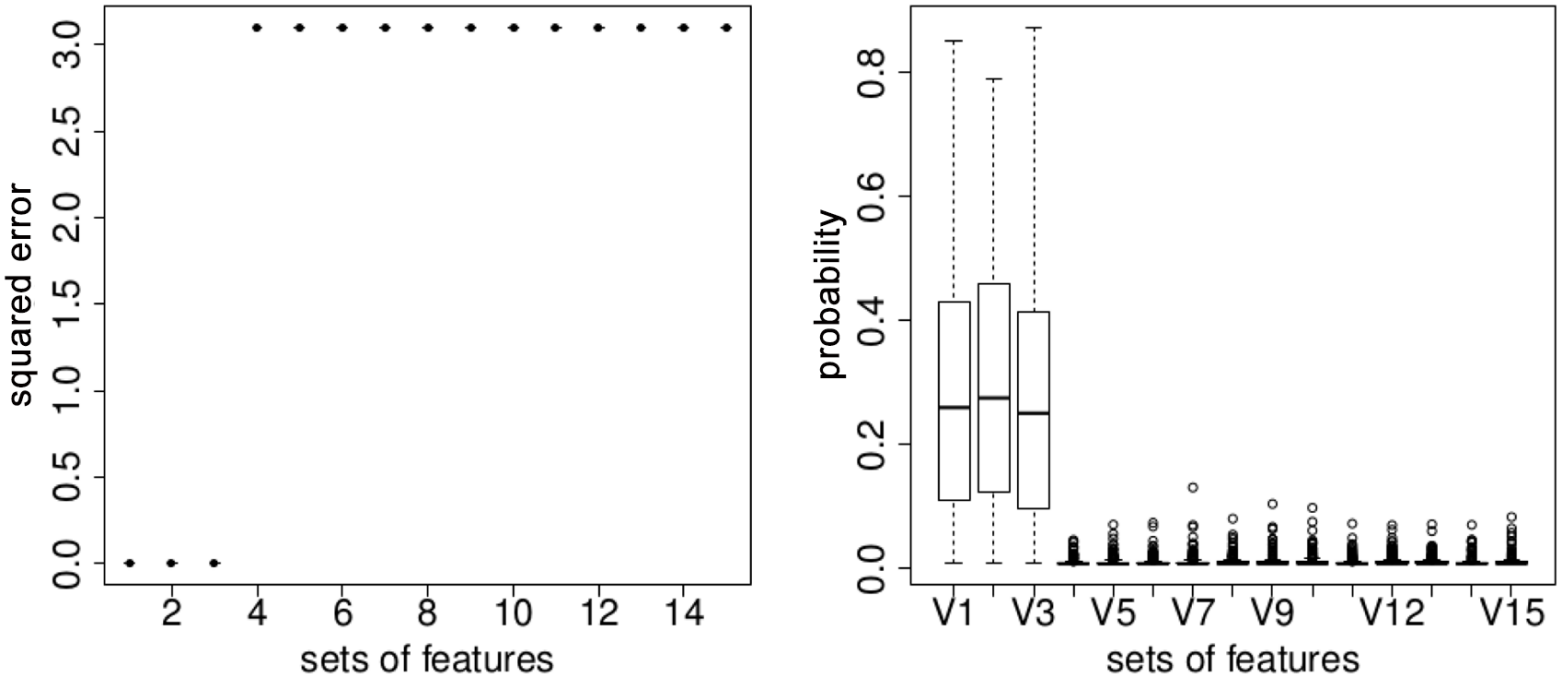
On the left: the squared error $\left\|\theta^{⋆}-{\hat{\theta }}^{n}\right\|$; on the right: probability estimated for each set of features by the EXP3 algorithm.

We have performed 200 Monte-Carlo simulations, and Fig 6 illustrates the result. The learning algorithm that we use inside the stochastic bandit to get the reward value, is the binary logistic regression, since the data is simulated from a well-specified logistic regression. On Fig 6, above we show the squared error $\left\|\theta^{⋆}-{\hat{\theta }}^{n}\right\|$, where *θ* ^⋆^ corresponds to the optimal model and ${\hat{\theta }}^{n}$ is the estimated vector of parameters. The models 1–3 are able to find the true model parameters (since the case is well-specified). Below, on Fig 6, we boxplot the probabilities estimated by the EXP3 and associated with each feature set. It is easy to see that the first three optimal feature sets have non-zero probabilities (around 0.3), and their sum goes to 1. The feature sets 4–15 have low probabilities, and aslo big squared errors.

This experiment shows that the stochastic bandit finds the optimal subsets of features, and that the estimated probability distribution corresponds to an optimal ranking.

### 5.3 MicrObese Data

We are motivated by a problem of patients stratification in order to choose an efficient appropriate personalized medical treatment. Our experiments are based on a recent French study [37] of gene-environment interactions carried out to understand the development of obesity. It was reported that the gut microbial gene richness can influence the outcome of a dietary intervention. A quantitative metagenomic analysis stratified patients into two groups: group with low gene gut flora count (LGC) and high gene gut flora count (HGC) group. The LGC individuals have a higher insulin-resistance and low-grade inflammation, and therefore the gene richness is strongly associated with obesity-driven diseases. The individuals from a low gene count group seemed to have an increased risk to develop obesity-related cardiometabolic risk compared to the patients from the high gene count group. It was shown [37] that a particular diet is able to increase the gene richness: an increase of genes was observed with the LGC patients after a 6-weeks energy-restricted diet. [38] conducted a similar study with Dutch individuals, and made a similar conclusion: there is a hope that a diet can be used to induce a permanent change of gut flora, and that treatment should be phenotype-specific.

The goal of our experiments is to predict to which group a patient belongs, and it is a binary classification problem. Another challenge is to find a compact subset of biomarkers which minimizes the prediction error.

#### 5.3.1 Brief Data Description

The MicrObese corpus contains meta-data (clinical data), gene expressions of adipose tissue, and gut flora metagenomic data. For each patient, we have the information to which class he or she belongs. There are two classes, high gene count (HGC) and low gene count (LGC) classes. Therefore, our problem is a binary prediction task from heterogeneous data. In general, 49 patients have been hired and examined at the Pitié-Salpêtrière hospital, Paris, France, but as to the genes of the adipose tissue, we dispose data for less patients, and not for all patients their class, LGC or HGC is provided. In our experiments we have access to 35 observations (patients) without missing data. To get rid of important noise, we run a significance test (Wilcoxon test), and we keep the variables for which the raw (not adjusted for the multiple hypothesis testing) *p*-values < 0.05.

We have 135 meta-parameters which can be divided into clinical parameters and alimentary patterns reflecting nourishing habits of the patients. The data set contains more than 42,000 genes of the adipose tissue, and the gut flora data contains counts for more than 3 million genes. The metagenomic matrix is quite sparse, and not all of the genes are significant. We have pre-selected 2,247 genes of gut flora and 8,220 genes of the adipose tissue for our further experiments. As to the clinical parameters, only 7 of them are significant enough (with respect to the LGC and HGC classes) to be considered in our experiments. So, the general number of parameters in our problem is 10,474 for 35 observations.

#### 5.3.2 Experiments on clinical, metagenomic, and transcriptomic data

Here we provide our results on the MicroObese data.

**Fig 7:**
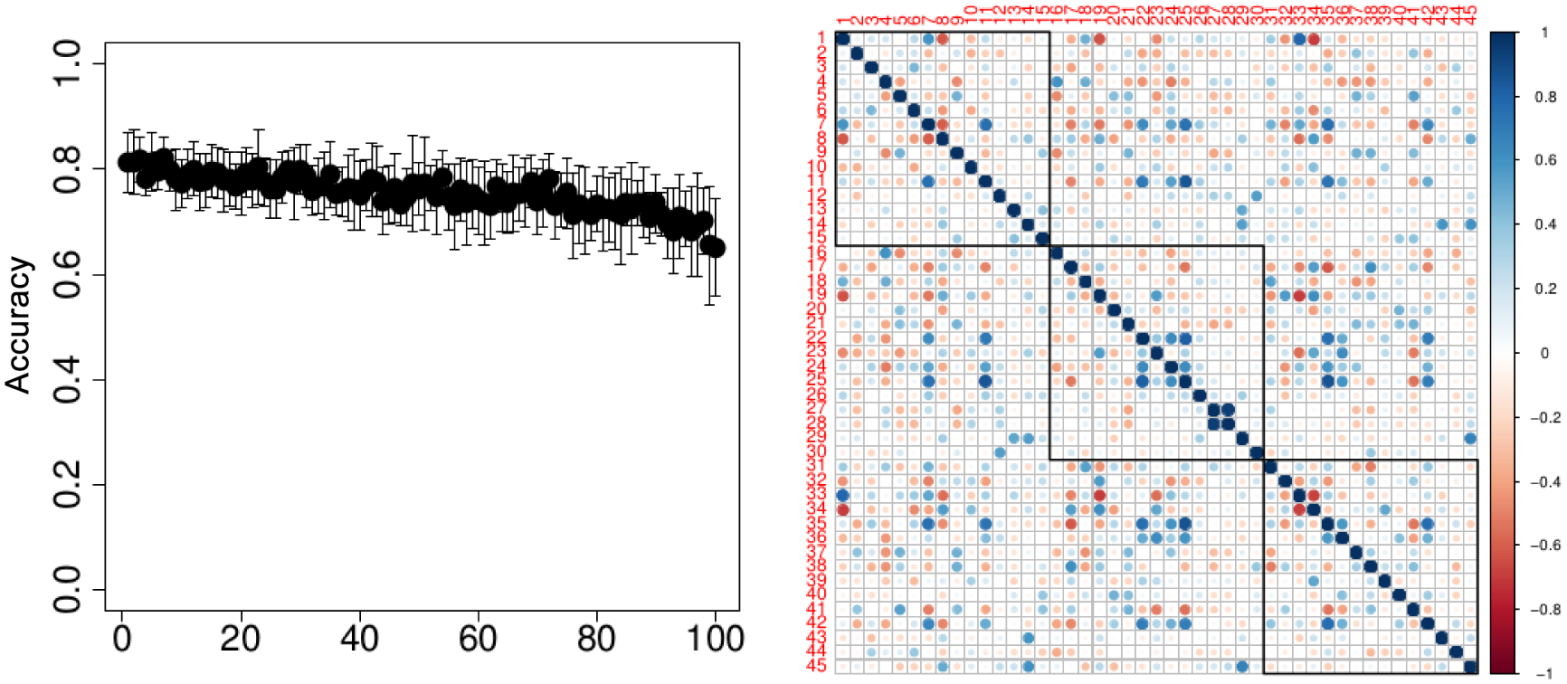
Experiments on the MicrObese data. On the left: accuracy for 100 feature sets ranked by the thresholding stochastic bandit; on the right: correlations between features of 3 feature sets with the highest ranking.

Fig 7 illustrates the results of the experiments, where we run 200 Monte-Carlo simulations. In each simulation, we have a different split of training and testing data. The number of all possible feature sets is very large, and it is intractable to rank all of them. Intuitively, most of them are not informative (we even know it, since we performed as well feature selection with a Kruskal-Wallis test, and a number of significant features is rather low).

The number of sets of features being tested is 100; we apply the thresholding stochastic bandit, introduced in Section 3, to select and and to rank the sets of features (the maximal number of iterations of EXP3 equals 3,000, and *α* = 0.27). Since the number of observations is limited to 35, we fix the number of parameters to be 30 (i.e. less than the number of observations). So, in order to find informative feature sets, we sample 100 features sets, each containing 30 features from about 10,500 possible parameters. Fig 7 above shows the accuracy of 100 ranked selected feature sets.

As expected, a number of feature sets lead to a high similar accuracy, although they consist of different features. This can be explained by existence of functionality categories of features, and we try to analyze the selected sets of parameters. Fig 7 below shows a correlation matrix between three highly ranked sets of features, where each set contains 15 features. The correlations within the same set are the blocks located on the diagonal, and marked with black squares. It is easy to see, however, that there are some strong, both positive and negative correlations between the blocks which are worth being studied and interpreted by biologists.

#### 5.3.3 Similarity and Stability of Biomarkers Selection on the Transcriptomic Data

Let us consider the similarity of the genes of the MicrObese adipose tissue, as well as stability and functional stability of the considered feature selection approaches.

**Fig 8:**
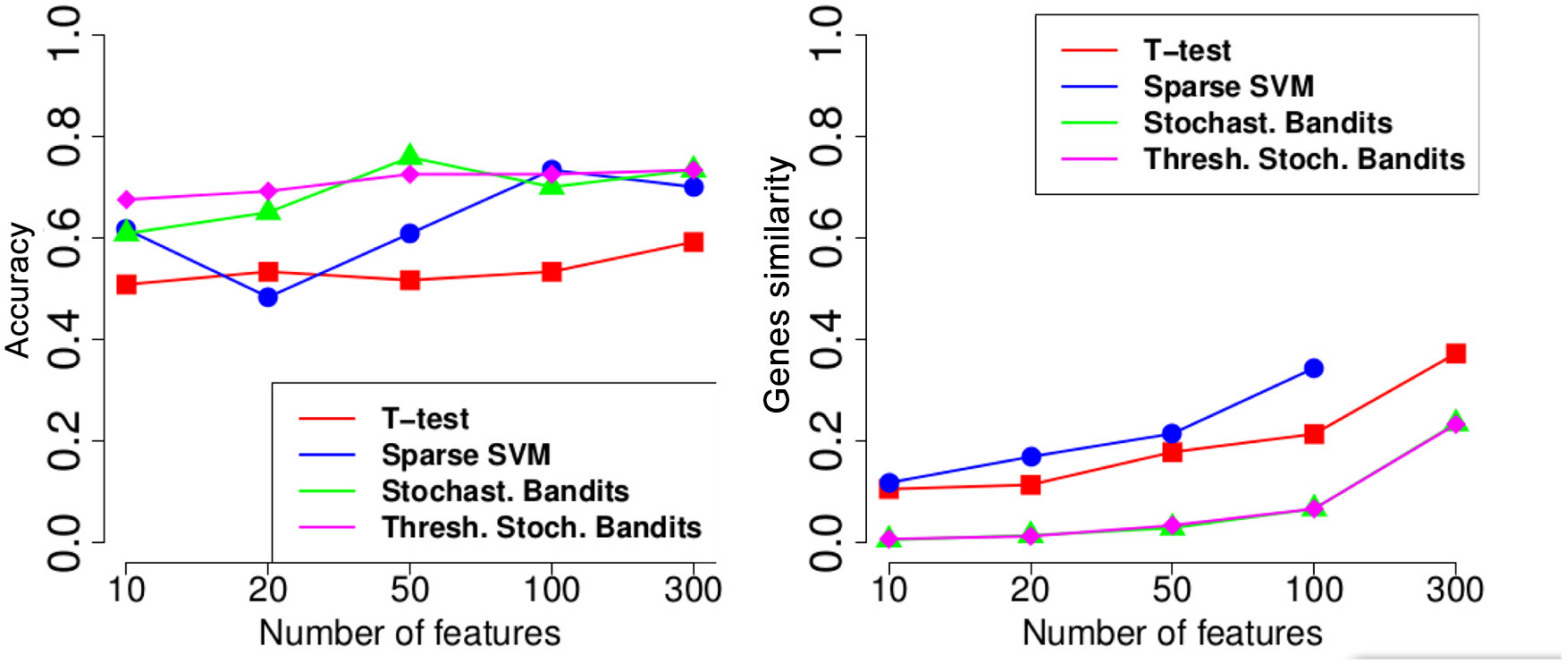
Experiments on the MicrObese transcriptomic data. On the left: accuracy; on the right: similarity on the level of separate genes.

**Fig 9:**
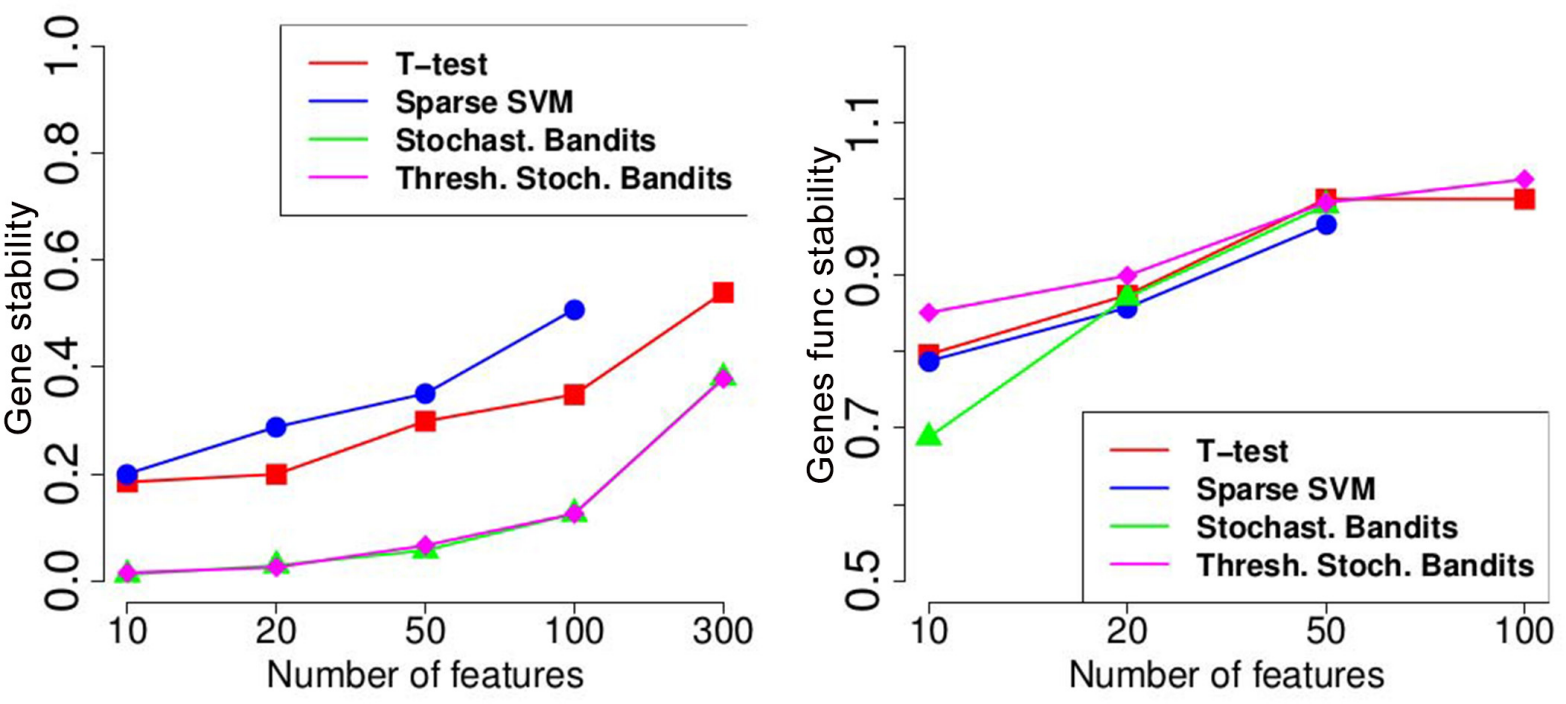
Experiments on the MicrObese transcriptomic data. On the left: stability; on the right: functional stability.

Fig 8 above shows the accuracy of the t-test, a sparse SVM [39], the stochastic bandit, and the thresholding stochastic bandit. Below, on Fig 8 we demonstrate the genes similarity for these methods. Fig 9 shows the stability on the genes level and the functional stability on the genes level.

It is interesting to see that the proposed stochastic thresholding bandit has a very poor similarity and stability values, since the features are randomly drawn. However, the functional stability of the thresholding bandit is higher compared to the state-of-the-art methods, since informative feature sets have high rankings. The accuracy of the novel thresholding bandit is quite high, and it significantly outperforms other methods, especially in cases where the number of selected features is small.

## 6 Conclusion

Biomarker selection is a crucial problem in biomedical high-dimensional applications. To find relationships between biomarkers, and to define functional families of features, is another problem related to feature selection. We have introduced a feature selection approach based on a sparse stochastic bandit, and we compared its predictive performance and stability to several state-of-the-art methods which are reported to be the most accurate and stable. We considered the newly introduced feature selection approach from cross-validation error rate and stability viewpoints, and our experiments on artificial and real data illustrate that the proposed approach is competitive. We also hope that functional classes of features learned from experiments, will provide additional knowledge and give rise to new hypotheses for biologists and physicians doing pre-clinical research.
